# Risk perception in the population living near the Turin municipal solid waste incineration plant: survey results before start-up and communication strategies

**DOI:** 10.1186/s12889-019-6808-z

**Published:** 2019-05-02

**Authors:** Antonella Bena, Martina Gandini, Ennio Cadum, Enrico Procopio, Giuseppe Salamina, Manuela Orengia, Elena Farina

**Affiliations:** 1 Department of Epidemiology, ASL TO3, Via Sabaudia 164, 10095 Grugliasco, TO Italy; 2 Environmental Epidemiological Unit, Regional Environmental Protection Agency, Piedmont Region, Via Pio VII 9, 10135 Torino, Italy; 3 Department of hygiene and health prevention, Environmental Health Unit, ATS Pavia, Pavia, Italy; 4 Prevention Department, ASL TO3, Collegno, Torino, Italy; 5 Prevention Department, ASL City of Turin, Torino, Italy

**Keywords:** Municipal solid waste incineration, Surveillance system, Risk perception, Scientific citizenry

## Abstract

**Background:**

The start-up of the Turin municipal solid waste incineration plant (2013) was accompanied by surveillance of health effects, which included a human biomonitoring campaign. Here we present the results of the risk perception survey of local residents before the plant went into operation.

**Methods:**

The survey sample was 394 local residents: 198 residing near the plant (exposed group) and 196 residing in an area distant from the plant site (unexposed group). The survey questionnaire investigated awareness of environmental and health issues, including a section on the perception of environmental health risks. Multivariate Poisson regressions were performed to determine the differences in risk perception between the two groups (exposed vs. unexposed).

**Results:**

The exposed group was more concerned about natural hazards (prevalence ratio [PR] 1.61; 95% confidence interval [CI] 0.99–2.61), anthropogenic hazards (PR 1.35; 95% CI 1.03–1.77), and waste management (PR 1.19; 95% CI 0.94–1.50). There were no significant differences in opinions about environmental pollution-related diseases between the two groups, though the exposed considered themselves to be at risk for developing these diseases. The survey population placed its trust more in health care providers than in any other category.

**Conclusions:**

The risk perception survey questionnaire yielded data that enabled a better understanding and interpretation of the social context: residents living near the incineration plant were more concerned than those living distant from it, especially about anthropogenic hazards. This information was subsequently incorporated into the design the communication tools.

**Electronic supplementary material:**

The online version of this article (10.1186/s12889-019-6808-z) contains supplementary material, which is available to authorized users.

## Background

Communication in human biomonitoring (HBM) has gained an increasingly important role, with numerous studies having identified tools that can effectively address related ethical issues and increase knowledge in communities where opposition to project location can be anticipated. HBM entails the collection of body fluids and generates complex results that need to be interpreted at both the individual and the collective level. The presence of outliers for substances with unknown health effects is a source of anxiety, however collective results may be difficult to communicate because they engender conflict due to a gap in risk perception between experts and the public. Effective public communication is further hindered by the diminishing trust in public authorities and institutions [1] further exacerbated by media keen on emphasizing controversy to create a story.

Effective risk communication rests on combining via rational decision-making processes technical expertise with public values and preferences [2]. The literature suggests that traditional strategies (meetings, newsletters, web sites) be coupled with modern communication tools (risk perception surveys, structured opportunities for stakeholder engagement and discussion with decision makers) [3]. Risk perception in HBM programmes can support local government agencies in evaluating the effectiveness of their prevention interventions [4]. Participation by institutional, programming, planning, and evaluation tools at various levels - European, national, regional and local - is envisaged [5].

HBM studies conducted in Italy have included communication strategies that combine traditional and innovative tools [4, 6]. One such study is SPoTT (SPoTT is the Italian acronym for Population Health Surveillance in the area of the Turin municipal solid waste incineration plant). The Turin plant went into operation in 2013. The plant site is located in an area heavily polluted with industrial emissions and consistent vehicular traffic. The local community had been initially involved in a participatory decision project to identify an area for locating the plant [7]. Other not previously considered sites were then identified and subjected to technical-environmental analyses. At the end of the decision process, another site was selected over the community’s original site of choice .

During plant construction, information campaigns, exploratory and public relations initiatives were implemented by the engineering company responsible for plant design and operation [8]; nonetheless, a strong opposition movement had gained momentum by the time the plant went into operation. The environmental agency ordered a HBM study and a multidisciplinary team designed SPoTT, which is a comprehensive HBM study [9]. A citizens association opposed to the plant proposed an alternative HBM study that would measure the presence of metals in children’s toenails. To date, neither the protocol nor the results of these analyses are available.

The project team decided to combine traditional communication strategies with modern risk communication tools to better interpret the local social context. The questionnaire for the SPoTT study was designed to gather information on work, lifestyle, health, and nutrition that would be useful for interpreting the biological data. The questionnaire also included a series of items investigating risk perception. Risk perception is a recognized element in defining suitable communication strategies and supporting effective prevention policies. Changes in risk perception after presentation of the HBM study results and of emission monitoring of the plant will be analyzed on conclusion of the SPoTT study after completion of the ongoing follow-up phases.

The effects of a community’s habituation to an incineration plant, in attitudes and risk perception [10], as well as prolonged concern about health effects and issues related to waste transport, have been documented [11]. This paper compares the results of a risk perception questionnaire two groups of residents completed before the incineration plant became operational and the alternatives for communication adopted as a consequence.

## Methods

### Communication tools

The SPoTT study employed several communication tools. The study protocol, approved by the local ethical committee, was available on the programme’s web site (www.dors.it/spott). A local monitoring committee, composed of local civil authorities, health and environmental technicians, held public meetings to illustrate the HBM programme. Upon enrolment, residents interested in participating in the study gave their written, informed consent after being informed about the study objectives and methods and personal data treatment. Participants could withdraw from the study at any time.

### The questionnaire

The SPoTT HBM questionnaire was administered by trained personnel to 394 local residents (age range, 35–69 years; resident for at least 5 years in one of the two study areas). The study population was divided into two groups: 196 residents living in an area where models estimated metal deposition < 0.007 mg/m^2^/year (unexposed area); 198 residents living in an area with estimated metal deposition > 0.014 mg/m^2^/year (exposure area). The recruitment percentage was 51.5% (394 of the 765 individuals contacted were enrolled). A CALPUFF Lagrangian particle software model that included the CALMET diagnostic wind model and the CALPOST post-processor was used to determine the fallout of the solid waste incinerator and obtain an estimate of metal deposition in the study area. The forecasting model was validated with the environmental monitoring station data for benzene, NO_2_, NO_x_ and PM_10_.

Study participants were randomly sampled from the municipal registry, stratified by sex and five-year age groups [9]. Due to the nature of the sampling strategy, participants recruited for the HBM were representative of the entire population for age and sex. The questionnaire included items on risk perception extracted from previous Italian HBM studies [12]. The English translation of the risk perception questionnaire is reported in the Additional file 1.

Two issues were investigated:

1. risk perception of environmental hazards
*Which of the following illnesses do you think are due to environmental pollution?*
*Do you think you are at risk of getting these diseases?*
  Diseases: allergies, acute respiratory diseases, chronic respiratory diseases, temporary organ damage, liver damage, cancer, leukaemia, congenital defects. The response was on a Likert-type scale from 1 to 4 where 1 indicated certain; 2 very probable; 3 quite probable; 4 not very probable; plus an additional response of “don’t know”.
*Which of these events concern or disturb you most?*
  Natural environmental calamities (floods, severe weather events, earthquakes) and anthropogenic hazards (noise, dangerous good transport, nuclear plant accidents, waste management, water and air pollution, hazardous industries, fires). The response was on a Likert-type scale from 1 to 4 where 1 indicated extremely; 2 very; 3 not very; 4 not at all; and an additional response of “don’t know”.
2. level of environmental information
  Do you feel well informed about environmental hazards?
  The response was on a Likert-type scale from 1 to 4 where 1 indicated extremely informed; 2 very informed; 3 not very informed; 4 not informed at all; plus an additional response of “don’t know”.
  Which of the following media do you prefer? National TV; local TV; local newspapers; Internet; other.
  Which source of information do you trust most? Local institutions and authorities; health care providers; environmental associations, other.

A large section of the HBM questionnaire included items investigating biological data, socio-demographic information, and state of health (sex, age, marital status, place of birth, level of education, having children, self-perceived health status). Part of this information was used in the analyses of the risk perception questionnaire to better identify differences in risk perception among categories.

### Statistical analysis

For each item, the absolute frequencies and percentages for each category of responses were calculated on the total sample of 394 individuals. To determine the differences in risk perception between the two groups (exposed vs. unexposed), Poisson regressions were performed to estimate prevalence ratios (PRs). Dependent variables were dichotomous: 1 denoted certain, very probable and 0 denoted quite probable, not very probable. The ‘don’t know’ responses were excluded from the analysis.

In a first set of analyses, concern about natural calamities, anthropogenic hazards, and waste management were dependent variables. The independent variables included in the multivariate model were: exposure area (exposed; unexposed), sex, age band (35–45, 46–55; 55+ years), place of birth (North; Centre-South; foreign), level of education (primary or lower secondary school education; upper secondary education; post secondary and tertiary education), marital status (single or widow/er); married/cohabiting, separated/divorced), with children (Yes; No), self-perceived health (good/poor state of health). Perceived health was self-assessed on a scale from 1 to 10, scores > 7 indicated a good state of health and scores ≤7 indicated a poor state of health. Occupational exposure (workplace exposure to dust, chemicals, radiation, etc.) that could have affected the risk perception pattern was not included in the analyses due to the low number of participants who reported such exposure.

In a second set of analyses, the variables related to opinions about environmental pollution-related diseases and the risk associated with developing them were dependent variables and the previous were independent variables. A 95% confidence interval (CI) was calculated. All statistical analyses were performed using SAS 9.2 software (SAS Institute Inc., Cary, NC, USA).

## Results

The distribution by sex and age was the same for both groups (Table 1), as expected according to the sampling strategy. The average age was 52 years in both groups. The unexposed group was composed of more respondents with a higher education level (high school or college), whereas the exposed group was made up of more married or cohabiting respondents and respondents with children.

**Table 1:** Characteristics of the sample (*N* = 394)

	Unexposed		Exposed		Total	
	No.	%	No.	%	No.	%
Sex (Males)	98	50.0	97	49.0	195	49.5
Place of birth						
North	120	61.2	128	64.6	248	62.9
Centre-South and Islands	61	31.1	64	32.3	125	31.7
Foreign	15	7.7	6	3.0	21	5.3
Marital Status						
Married/cohabiting	151	77.4	163	82.3	314	80.0
Separated/divorced	11	5.6	14	7.0	25	6.4
Single/Widow/er	33	16.9	21	10.6	54	13.7
Children (Yes)	149	76.0	166	83.8	315	79.9
Age class (yrs)						
30–45	58	29.6	57	28.8	115	29.2
45–55	61	31.1	63	31.8	124	31.5
> 55	77	39.3	78	39.4	155	39.3
Level of Education*						
Primary/lower secondary	68	35.1	91	46.0	159	40.5
Upper secondary	79	40.7	86	43.4	165	42.1
Post secondary	47	24.2	21	10.6	68	17.3
Self-perceived health						
Good	132	67.4	141	71.4	273	69.3
Poor	64	32.7	57	28.8	121	30.7

*Significant difference between exposed and unexposed (*p*-value of chi-square test < 0.05)

**Fig. 1:**
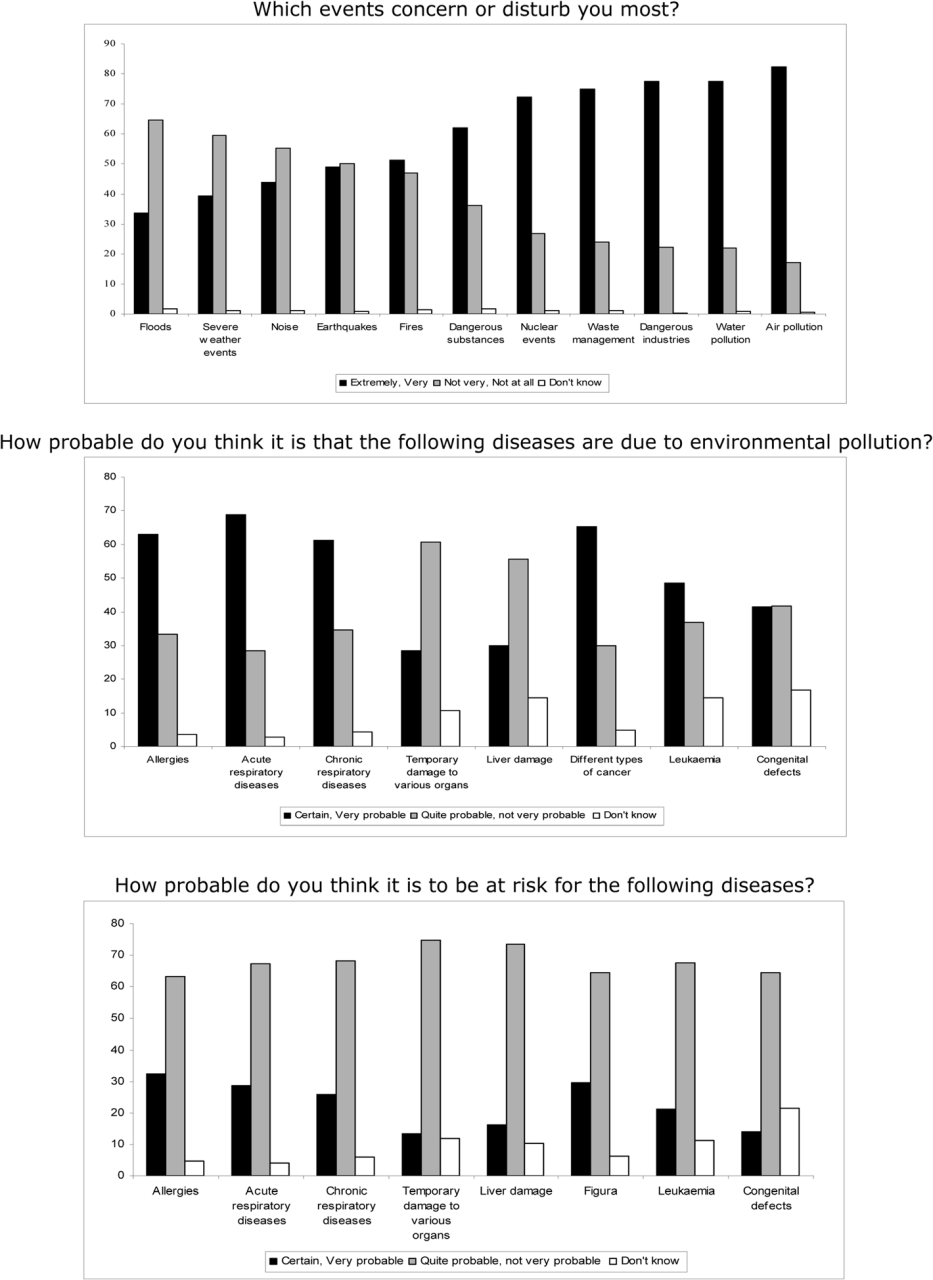
Risk perception of hazards and diseases (394 respondents)

Anthropogenic hazards were of greater concern than natural calamities (Fig. 1). Overall, air pollution was of greatest concern expressed by the sample: more than 80% of the respondents stated they were extremely or very worried about air pollution. This was followed by concern about waste management, with more than 70% of respondents stating they were extremely or very concerned about it.

The exposed group was more concerned than the unexposed group about natural and anthropogenic hazards, after adjusting for all the other variables included in the model (Table 2). For the other variables, the prevalence ratio for anthropogenic hazards was almost always near 1, indicating no differences between categories. As regards natural calamities, the results showed that the probability of being concerned was almost three times higher for women than for men (PR 2.77, CI 1.62–4.73). As compared with respondents aged over 55 years, concern was lower among the 35–45 age group (PR 0.50, CI 0.25–1.03). Over 60% of respondents stated that allergies, acute and chronic respiratory diseases, as well as different types of cancer were probably associated with environmental pollution. In contrast, only 25–30% of respondents were certain or considered it as very probable that they were at risk of developing these diseases (Fig. 1).

**Table 2:** Differences in perception of natural and anthropogenic hazards and waste management: Prevalence ratio adjusted for all other variables (CI 95%)

	Natural hazards	Anthropogenic hazards	Waste management
Exposure (vs. Unexposed)			
Exposed	1.61 (0.99–2.61)	1.35 (1.03–1.77)	1.19 (0.94–1.50)
Sex (vs. Males)			
Females	2.77 (1.62–4.73)	1.20 (0.92–1.58)	1.10 (0.87–1.40)
Marital Status (vs. Married/cohabiting)			
Single/Widow/er	1.24 (0.61–2.50)	1.01 (0.66–1.56)	1.02 (0.70–1.47)
Separated/divorced	0.76 (0.30–1.92)	0.94 (0.55–1.61)	0.82 (0.49–0.38)
Self-perceived health (vs. Good)			
Poor	1.31 (0.81–2.10)	1.04 (0.78–1.39)	0.99 (0.77–1.28)
Place of birth (vs. North)			
Centre-South and Islands	1.31 (0.79–2.18)	0.98 (0.72–1.34)	1.04 (0.80–1.37)
Foreign	1.08 (0.33–3.54)	1.08 (0.59–1.97)	0.92 (0.53–1.60)
With children (vs. No)			
Yes	1.10 (0.54–2.23)	1.09 (0.74–1.59)	1.08 (0.78–1.51)
Age class (vs. > 55 yrs)			
(30.45]	0.50 (0.25–1.03)	0.82 (0.57–1.18)	0.94 (0.69–1.28)
(45.55]	0.99 (0.59–1.67)	1.19 (0.88–1.62)	1.09 (0.83–1.44)
Level of Education (vs. Primary/lower secondary)			
Upper secondary	0.89 (0.54–1.48)	1.02 (0.76–1.37)	0.99 (0.76–1.29)
Post secondary	0.44 (0.16–1.16)	0.89 (0.58–1.36)	1.08 (0.76–1.54)

When we controlled for all the other variables, we noted that the exposed group thought it more probable that the diseases were due to pollution, even though the prevalence ratio was not statistically significant (Table 3). Women in general considered it more probable than men that the diseases might be due to pollution, particularly allergies, liver damage, cancer, leukaemia, and congenital defects. Respondents with children stated that they thought it more probable that allergies and chronic respiratory diseases were caused by air pollution. Respondents with a higher level of education thought it more probable that, except for cancer the other diseases might be due to pollution.

**Table 3:** Differences in perception of risk of contracting an environmental pollution-related disease and risk of disease: Prevalence ratio adjusted for all other variables (CI 95%)

	Allergies	Acute respiratory diseases	Chronic respiratory diseases	Temporary organ damage	Liver damage	Cancer	Leukaemia	Congenital defects
How probable do you think it is that the following diseases are due to environmental pollution?								
Exposure (vs. Unexposed)								
Exposed	1.02 (0.79–1.31)	1.09 (0.85–1.40)	1.09 (0.84–1.42)	1.15 (0.79–1.69)	1.19 (0.82–1.73)	1.12 (0.87–1.44)	1.36 (1.01–1.83)	1.29 (0.94–1.78)
Sex (vs. Males)								
Females	1.20 (0.92–1.55)	1.03 (0.81–1.32)	1.06 (0.82–1.38)	1.02 (0.69–1.49)	1.15 (0.79–1.67)	1.16 (0.90–1.49)	1.18 (0.88–1.59)	1.18 (0.86–1.63)
Marital Status (vs. Married/cohabiting)								
Single/Widow/er	1.12 (0.75–1.65)	1.17 (0.80–1.70)	1.14 (0.76–1.72)	1.13 (0.62–2.07)	1.30 (0.75–2.25)	1.02 (0.69–1.51)	1.13 (0.73–1.76)	1.14 (0.71–1.84)
Separated/divorced	1.00 (0.61–1.66)	1.10 (0.68–1.78)	1.02 (0.61–1.72)	1.02 (0.47–2.23)	0.55 (0.20–1.51)	0.81 (0.46–1.44)	0.94 (0.53–1.67)	0.87 (0.45–1.67)
Self-perceived health (vs. Good)								
Poor	1.08 (0.83–1.42)	0.97 (0.75–1.27)	0.91 (0.68–1.22)	0.87 (0.57–1.33)	0.88 (0.59–1.33)	0.99 (0.75–1.30)	1.07 (0.78–1.46)	1.08 (0.77–1.52)
Place of birth (vs. North)								
Centre-South and Islands	0.97 (0.72–1.31)	1.06 (0.80–1.42)	1.08 (0.79–1.46)	1.37 (0.89–2.11)	1.27 (0.83–1.93)	0.99 (0.74–1.33)	1.05 (0.75–1.46)	1.02 (0.70–1.48)
Foreign	0.87 (0.48–1.58)	1.04 (0.61–1.79)	0.99 (0.54–1.14)	1.16 (0.50–2.73)	0.94 (0.37–2.38)	0.76 (0.39–1.51)	0.70 (0.31–1.61)	0.84 (0.37–1.95)
With children (vs. No)								
Yes	1.23 (0.86–1.76)	1.04 (0.74–1.46)	1.27 (0.87–1.84)	1.16 (0.67–2.03)	1.07 (0.63–1.82)	1.16 (0.80–1.66)	1.28 (0.84–1.97)	1.19 (0.75–1.88)
Age class (vs. > 55 yrs)								
(30.45]	0.91 (0.65–1.28)	1.00 (0.73–1.38)	1.06 (0.76–1.49)	0.85 (0.52–1.40)	0.77 (0.48–1.25)	1.12 (0.81–1.55)	0.93 (0.64–1.37)	1.04 (0.69–1.56)
(45.55]	1.08 (0.79–1.46)	1.09 (0.81–1.46)	1.04 (0.76–1.42)	0.81 (0.51–1.27)	0.81 (0.52–1.27)	1.14 (0.84–1.54)	0.93 (0.66–1.32)	0.81 (0.55–1.19)
Level of Education (vs. Primary/lower secondary)								
Upper secondary	1.21 (0.90–1.62)	1.16 (0.88–1.53)	1.35 (1.00–1.82)	1.22 (0.73–2.04)	1.40 (0.91–2.14)	1.01 (0.76–1.34)	1.17 (0.84–1.63)	1.15 (0.81–1.64)
Post secondary	1.29 (0.88–1.90)	1.11 (0.77–1.62)	1.30 (0.88–1.93)	1.31 (0.74–2.33)	1.22 (0.68–2.19)	0.99 (0.67–1.45)	1.09 (0.69–1.73)	1.00 (0.61–1.65)
How probable do you think it is to be at risk for the following environmental pollution-related diseases?								
Exposure (vs. Unexposed)								
Exposed	1.61 (1.11–2.33)	1.63 (1.10–2.41)	1.56 (1.04–2.35)	3.66 (1.86–7.19)	2.95 (1.64–5.32)	3.32 (2.11–5.22)	4.55 (2.54–8.14)	6.31 (2.81–14.16)
Sex (vs. Males)								
Females	1.52 (1.06–2.20)	1.39 (0.94–2.04)	1.43 (0.96–2.15)	1.49 (0.84–2.64)	1.33 (0.79–2.25)	1.14 (0.78–1.67)	1.28 (0.80–2.03)	1.19 (0.67–2.10)
Marital Status (vs. Married/cohabiting)								
Single/Widow/er	0.98 (0.56–1.71)	0.81 (0.43–1.52)	0.68 (0.34–1.38)	1.03 (0.41–2.59)	1.25 (0.52–2.99)	0.97 (0.50–1.87)	0.78 (0.34–1.81)	1.55 (0.65–3.71)
Separated/divorced	0.55 (0.24–1.26)	0.68 (0.29–1.57)	0.79 (0.34–1.83)	1.34 (0.51–3.49)	1.08 (0.42–2.80)	0.94 (0.45–1.97)	0.90 (0.38–2.12)	0.91 (0.32–2.63)
Self-perceived health (vs. Good)								
Poor	1.66 (1.15–2.39)	1.35 (0.91–2.00)	1.54 (1.02–2.32)	0.81 (0.43–1.53)	1.06 (0.61–1.84)	1.17 (0.78–1.74)	1.04 (0.64–1.68)	1.34 (0.76–2.36)
Place of birth (vs. North)								
Centre-South and Islands	1.63 (1.09–2.44)	1.33 (0.87–2.03)	1.22 (0.78–1.91)	1.81 (0.98–3.34)	1.47 (0.85–2.57)	1.16 (0.76–1.75)	1.36 (0.84–2.21)	1.41 (0.78–2.56)
Foreign	1.03 (0.41–2.58)	0.21 (0.03–1.53)	0.22 (0.03–1.57)	0.67 (0.09–5.04)	0.50 (0.07–3.72)	0.72 (0.22–2.32)	0.74 (0.17–3.10)	0.55 (0.07–4.10)
With children (vs. No)								
Yes	1.27 (0.75–2.16)	1.20 (0.68–2.09)	1.23 (0.68–2.22)	1.29 (0.54–3.07)	1.90 (0.75–4.81)	1.35 (0.74–2.48)	1.16 (0.56–2.40)	1.28 (0.53–3.10)
Age class (vs. > 55 yrs)								
(30.45]	2.06 (1.26–3.36)	1.41 (0.87–2.29)	1.40 (0.84–2.33)	1.11 (0.54–2.26)	1.30 (0.67–2.52)	1.02 (0.62–1.68)	0.94 (0.51–1.71)	0.94 (0.44–2.00)
(45.55]	1.89 (1.21–2.94)	1.10 (0.69–1.75)	1.09 (0.67–1.77)	0.77 (0.39–1.53)	0.90 (0.49–1.66)	0.96 (0.62–1.49)	0.89 (0.53–1.49)	0.88 (0.46–1.70)
Level of Education (vs. Primary/lower secondary)								
Upper secondary	1.31 (0.88–1.94)	1.21 (0.80–1.84)	1.05 (0.68–1.63)	1.32 (0.71–2.43)	0.69 (0.40–1.21)	0.86 (0.57–1.30)	0.83 (0.51–1.37)	0.74 (0.40–1.37)
Post secondary	0.87 (0.47–1.60)	0.90 (0.48–1.71)	0.93 (0.49–1.76)	1.30 (0.52–3.25)	0.47 (0.17–1.25)	0.78 (0.41–1.48)	0.67 (0.30–1.50)	0.82 (0.32–2.07)

The exposed group perceived themselves to be much more at risk than the unexposed group, with prevalence ratio of > 1.5 and statistically significant for all diseases. Again, women stated they felt more at risk than men. Respondents with children considered themselves more at risk than those without children. No clear pattern emerged for educational level. Respondents declaring a poor state of health felt more at risk for developing disease, except temporary organ damage, than those who declared a good state of health. As compared with respondents born in the north of the country, those born elsewhere felt less at risk for developing disease and thought they were sufficiently well informed about environmental risks: the prevalence was slightly higher in the exposed compared to the unexposed group (86.4% vs. 78.5%). As regards media sources of information, the main source was national television (47%). Finally, 39% of respondents thought that health care providers were the most reliable source of information, followed by environmental associations (27%), and local authorities and agencies (25%).

## Discussion

For most of the aspects under investigation, the residents living closer to the incineration plant expressed greater concern than those living far from it. Anthropogenic hazards generated more concern than natural hazards. Our results are shared by a previous study conducted in Italy: people living in industrial areas are noted to have greater risk perception than those living in areas where natural hazards are present [4]. Salvati et al. [13] found that people in Italy feel more vulnerable to anthropogenic than natural risk. Public acceptance of anthropogenic risk is influenced by trust and local experience. Furthermore, it is conditioned and constantly revised by information from multiple sources, including the media, and by the influence of peers and others: communication plans must have reliable tools to support such elements. In line with the literature, we found an inverse proportion between greater risk perception and greater distance from the incineration plant [10, 14]. Indeed, risk perception is the most important predictor of attitudes towards an incineration plant: when negative, such attitudes are not simply due to personal interests (NIMBY syndrome) but rather are related to the perception of unequal distribution of the consequences and the way decision processes are managed. This is especially true for newly constructed plants, as seen also in Turin, where the concern of the residents living closest to the plant was high due to the injustice they felt because their choice of incineration site was overridden by extraneous forces.

The situation is more complex concerning plants that have been in operation for many years. In Modena, for example, where the incineration plant has been operational for more than 30 years, no relationship was found between residence near the plant and citizens’ attitudes toward it [6]. Cavazza et al. found that the level of risk perception, the amount of knowledge, the degree of involvement, and the level of cognitive and affective ambivalence are important aspects that define the attitude of the local communities towards a plant. Turin institutions need to take into account all of these processes and adopt continuous communication planning not only during plant construction or after a problem occurs, but also during plant operation in order to foster trust, participation and active involvement of the community, particularly the residents living near the incineration plant site. The questionnaire investigating risk perception was repeated during the latest follow-up of the HBM study: analysis of the responses, which is ongoing, will allow to detect effectiveness of actions undertaken.

There were no significant differences between the two groups regarding health risk perception of environmental pollution-related diseases. Nevertheless, only the exposed believed they were at risk for developing such diseases. This reveals an overall concern about environmental issues, but with differential perception according to the area of residence. The diseases that trigger a response of greater concern are those related to cancer, respiratory problems, and allergies. The concern about congenital defects was significantly higher than about other diseases. This points toward one of the major problems of risk perception: the not always founded relationship between what constitutes a source of anxiety for the community and the actual existence of a health risk. Indeed, congenital defects are rare events, much more rare than other diseases. Furthermore the literature indicates limited evidence of increased risk for certain types of cancer [15] and for congenital defects as a whole [16]. These studies, however, mainly refer to old plants and have several methodological flaws which restrict the validity of the results (exposure assessment is often poor; the analyses are at an ecological level, with no control for potential individual confounders or reference to few individuals; little information is given on confounding factors). Studies conducted in Italy have indicated an increase in preterm birth [17, 18] and effects of air pollution on respiratory diseases among men [19]. To summarize, the order of magnitude of the risks described in the literature could differ from what people perceive.

Other examples are documented in the study by Salvati et al. that showed that perception of the threat posed by geohydrological events does not effectively match the risk posed by landslides and floods to the local population [13]. In such cases promotion of a scientific citizenry could set the basis for everyone, even those with a lower level of education, to understand the main scientific issues and form an educated, informed opinion. As regards the Turin incineration plant, plant construction was accompanied by information campaigns, exploratory and public relations initiatives; however, interruption of the participatory decision-making process for identifying the plant site disoriented the local community and fuelled opposition to its construction [8]. The HBM study will need to be accompanied by periodic reporting of the results, both to those directly involved in the HBM and to the overall community. A final crucial point is to ensure efficient knowledge transfer from the HBM study to local policy makers in order to facilitate an evidence-based risk governance.

Questionnaire respondents expressed the highest percentage of trust (39%) in health care providers; nonetheless, a general lack of trust can be observed in the percentage of responses classified as “other”, especially among the exposed group (28% vs. 9%). Higher trust in environmental associations might have been expected, given that their websites disseminated numerous reports opposing plant construction during the period when the interviews were conducted. In addition, a citizens movement openly protesting against plant construction organised and advertised an alternative study that would purportedly demonstrate metal deposition in children’s nails.

Only a quarter of the respondents stated that they placed trust in the local institutions, with a slightly lower percentage noted for the exposed group (21% vs. 29%). Such mistrust was probably also a result of the participatory decision process which, as mentioned above, was started but then stopped when construction on the plant was begun. Since the trust of local communities in the public institutions remains a fundamental problem to be addressed, putting in place actions to acquire the public’s trust and keep it is of paramount importance [20]. Nevertheless, there are academics who relativise the importance of risk communication, considering it more important to define rules and procedures that are well-understood and accepted by local communities, enabling effective participation and clear information [21]. The lack of trust could paradoxically have a positive effect since it may stimulate critical thinking and, consequently, more careful analysis of the problems and possible solutions. It can be assumed that most people have limited technical knowledge and that only the really motivated will take an interest in the topic and seek further information. Therefore, the most critical issue in the communication process is not the transmission of a large quantity of information, but rather the reputation of the sources. In the Turin case-study, the level of trust in health care providers, although better than the others, could be increased over time: objectives, timing, limits and uncertainties must be clearly defined, keeping the promises made and reporting on the problems as they arise.

## Conclusions

The risk perception questionnaire completed by the citizens participating in the Turin HBM project provided a better understanding and interpretation of the social context in question. The residents living close to the incineration plant were more concerned than those living more distant from the plant, especially with regard to anthropogenic hazards. Indeed, as compared with the unexposed group, more people in the exposed group stated that certain diseases were related to environmental pollution and they perceived themselves as being at greater risk for developing pollution-related diseases.

The information gleaned from the questionnaire was then incorporated into the design of the communication tools. From the outset, a communication plan was developed which, based on international guidelines [22], identified the relevant audience, the most appropriate strategies and tools to ensure the transferability of useful information to all subjects and ensure transparency of the message transferred to population. Communication with local policy-makers takes place in the public arena (in the presence of the local community) within the scope of a Local Control Committee which meets regularly. There is an area on the regional health documentation centre website (www.dors.it/spott) where the documents concerning the programme are posted in a timely manner, as well as a summary of the literature on solid waste incineration plants and their effects on health. Valuable information can also be sourced via videos on YouTube. There is an e-mail address through which citizens can request clarification. The authors think that these measures might contribute to: decrease the difference in risk perception between the two groups; increase trust in health care providers and local authorities. The change in risk perception after presentation of the results of the HBM study and monitoring of the plant emissions will be analyzed after completion of the ongoing follow-up phases.

## Additional file

Additional file 1: Risk perception questionnaire. Contains all the questions regarding the risk perception section. (DOCX 17 kb)

## Abbreviations

CI: Confidence Interval
HBM: Human Biomonitoring
PR: Prevalence Ratio
RP: Risk Perception
SPoTT: Population health Surveillance in the area of the Turin municipal solid waste incineration plant
