# Supplementary material for: Risk perception in the population living near the Turin municipal solid waste incineration plant: survey results before start-up and communication strategies
Source: BMC Public Health. 2019 May 2;19:483. doi: 10.1186/s12889-019-6808-z (PMC6498555; doi:10.1186/s12889-019-6808-z)
Supplement: Supplementary file 1 — Risk perception questionnaire. Contains all the questions regarding the risk perception section. (DOCX 17 kb) [file 12889_2019_6808_MOESM1_ESM.docx]

**RISK PERCEPTION QUESTIONNAIRE**

WHICH OF THE FOLLOWING ILLNESSES DO YOU THINK ARE DUE TO ENVIRONMENTAL POLLUTION?

|  | Certain | Very probable | Quite probable | Not very probable | Don’t know |
| --- | --- | --- | --- | --- | --- |
| Allergies | \|__\| | \|__\| | \|__\| | \|__\| | \|__\| |
| Acute respiratory diseases | \|__\| | \|__\| | \|__\| | \|__\| | \|__\| |
| Chronic respiratory diseases | \|__\| | \|__\| | \|__\| | \|__\| | \|__\| |
| Temporary organ damage | \|__\| | \|__\| | \|__\| | \|__\| | \|__\| |
| Liver damage | \|__\| | \|__\| | \|__\| | \|__\| | \|__\| |
| Cancer | \|__\| | \|__\| | \|__\| | \|__\| | \|__\| |
| Leukaemia | \|__\| | \|__\| | \|__\| | \|__\| | \|__\| |
| Congenital defects | \|__\| | \|__\| | \|__\| | \|__\| | \|__\| |

DO YOU THINK YOU ARE AT RISK OF GETTING THESE DISEASES?

|  | Certain | Very probable | Quite probable | Not very probable | Don’t know |
| --- | --- | --- | --- | --- | --- |
| Allergies | \|__\| | \|__\| | \|__\| | \|__\| | \|__\| |
| Acute respiratory diseases | \|__\| | \|__\| | \|__\| | \|__\| | \|__\| |
| Chronic respiratory diseases | \|__\| | \|__\| | \|__\| | \|__\| | \|__\| |
| Temporary organ damage | \|__\| | \|__\| | \|__\| | \|__\| | \|__\| |
| Liver damage | \|__\| | \|__\| | \|__\| | \|__\| | \|__\| |
| Cancer | \|__\| | \|__\| | \|__\| | \|__\| | \|__\| |
| Leukaemia | \|__\| | \|__\| | \|__\| | \|__\| | \|__\| |
| Congenital defects | \|__\| | \|__\| | \|__\| | \|__\| | \|__\| |

WHICH OF THESE EVENTS CONCERN OR DISTURB YOU MOST?

| Natural environmental calamities | |  |  |  |  |
| --- | --- | --- | --- | --- | --- |
|  | Extremely | Very | Not very | Nota t all | Don’t know |
| Severe weather events | \|__\| | \|__\| | \|__\| | \|__\| | \|__\| |
| Earthquakes | \|__\| | \|__\| | \|__\| | \|__\| | \|__\| |
| Floods | \|__\| | \|__\| | \|__\| | \|__\| | \|__\| |
| Other | \|__\| | \|__\| | \|__\| | \|__\| | \|__\| |
|  |  |  |  |  |  |
| Anthropogenic hazards | |  |  |  |  |
|  | Extremely | Very | Not very | Nota t all | Don’t know |
| Noise | \|__\| | \|__\| | \|__\| | \|__\| | \|__\| |
| Dangerous good transport | \|__\| | \|__\| | \|__\| | \|__\| | \|__\| |
| Nuclear plant accidents | \|__\| | \|__\| | \|__\| | \|__\| | \|__\| |
| Waste management | \|__\| | \|__\| | \|__\| | \|__\| | \|__\| |
| Air pollution | \|__\| | \|__\| | \|__\| | \|__\| | \|__\| |
| Hazardous industries | \|__\| | \|__\| | \|__\| | \|__\| | \|__\| |
| Fires | \|__\| | \|__\| | \|__\| | \|__\| | \|__\| |
| Water pollution | \|__\| | \|__\| | \|__\| | \|__\| | \|__\| |
| Other | \|__\| | \|__\| | \|__\| | \|__\| | \|__\| |

DO YOU FEEL WELL INFORMED ABOUT ENVIRONMENTAL HAZARDS?

- 1. Extremely informed
  2. Very informed
  3. Not very informed
  4. Not informed at all
  5. Don’t know

WHICH OF THE FOLLOWING MEDIA DO YOU PREFER?

1. National TV
2. Local TV
3. Local newspapers
4. Internet
5. Other

WHICH SOURCE OF INFORMATION DO YOU TRUST MOST?

1. Local institution and authorities
2. Health care providers
3. Environmental associations
4. Other
